# Exploration of the Potential Mechanism of Metal Ion Transport‐Related Genes in Myocardial Infarction Based on Transcriptomics

**DOI:** 10.1111/jcmm.71134

**Published:** 2026-04-09

**Authors:** Xiaowei Zhou, Fanyan Luo, Meihong Yu, Yao Deng, Kaixuan Li

**Affiliations:** ^1^ The Department of Cardiovascular Surgery Xiangya Hospital, Central South University Changsha Hunan Province China; ^2^ National Clinical Research Center for Geriatric Disorders Xiangya Hospital, Central South University Changsha Hunan China; ^3^ Department of Gastroenterology The Second Xiangya Hospital of Central South University Changsha China; ^4^ Research Center of Digestive Disease Central South University Changsha China

**Keywords:** hub genes, metal ion transport, myocardial infarction, transcriptome

## Abstract

Myocardial infarction (MI), a severe coronary artery disease manifestation, is a top global cause of death. Abnormal metal ion transport (MIT) is key to MI pathological progression, and this study explores MIT's molecular characteristics/regulatory mechanisms in MI via transcriptome and bioinformatics data. Differentially expressed genes (DEGs) in MI were screened, and their intersection with MIT‐related genes (MITRGs) identified candidate genes. Protein–protein interaction (PPI) network analysis, expression validation, gene set enrichment, immune infiltration analyses, and regulatory network construction were used to find hub genes and mechanisms. Through algorithmic analysis combined with gene expression validation, Ace, Igf1, Lgals3, and Serpine1 were identified as hub genes. These hub genes are mainly involved in metabolic pathways such as the TCA cycle and lysosomes. In the MI group, the infiltration of innate immune cells was increased, while that of adaptive immune cells was decreased. Additionally, Serpine1, Lgals3, Ace, and Igf1 showed a significant positive correlation with macrophages and monocytes. Various potential drugs were predicted for these hub genes. Molecular docking revealed strong binding capacities between these human hub genes and their corresponding drugs. Through bioinformatics analysis, this study identified four hub genes closely associated with MIT in MI, providing a new research basis for the mechanism of MI.

## 1 Introduction

Myocardial infarction (MI) is a cardiovascular disease characterised by high morbidity and mortality [1]. It leads to various cardiac pathophysiological changes, including ischemia/reperfusion injury, inflammation, fibrosis, and ventricular remodelling, posing a significant threat to global health [2]. MI can damage myocardial cells and lead to the formation of fibrous scars, which may eventually result in heart failure [3]. In the damaged myocardium (a site undergoing repair post‐MI), the phases of inflammation, fibrosis, and angiogenesis—three key processes in myocardial repair—overlap. After MI, under ischemic and hypoxic conditions, myocardial cells undergo a wave of apoptosis within hours to days. The damaged tissue triggers an inflammatory response, leading to the infiltration of inflammatory cells and the release of proinflammatory cytokines and chemokines, thereby promoting the formation of granulation tissue [4]. Current treatments for MI include thrombolysis and surgical interventions such as percutaneous coronary intervention (PCI) and coronary artery bypass grafting (CABG). However, vascular recanalisation achieved by these treatments can lead to reperfusion injury, exacerbating myocardial damage and microcirculatory disorders, and further impairing cardiac function [5]. Although clinical reperfusion therapies and conventional pharmacological interventions have improved acute‐phase survival rates and short‐term prognosis, their ability to provide lasting improvement in cardiac function or reverse pathological progression remains limited [6]. Therefore, to address these limitations, there is an urgent need to identify novel, highly specific hub genes and molecular pathways which could serve as critical therapeutic targets for MI.

Metal Ion Transport (MIT) refers to the transmembrane transport process of metal ions (such as Na^+^, K^+^, Ca^2+^, etc.) within the body or within cells [7]. In recent years, abnormalities in MIT—one of the key biological processes involved in the pathological progression of MI—have gradually garnered extensive attention from researchers [8]. Under ischemic and hypoxic conditions in myocardial tissue, the homeostasis of metal ions (e.g., iron and calcium) between the intracellular and extracellular spaces is disrupted, leading to a series of imbalances in signal transduction and subsequent cellular dysfunction [9]. For instance, disturbances in calcium ion transport—as a core component of MIT—directly affect the contractile function and electrophysiological stability of myocardial cells. Additionally, cell death modalities such as cuproptosis and ferroptosis—which are tightly linked to abnormal metal ion metabolism (e.g., copper and iron homeostasis)—have been identified in MI. As such, the role of abnormal MIT in mediating the pathogenesis of MI has increasingly garnered attention [10, 11, 12]. Therefore, an in‐depth exploration of the relationship between MIT and MI is expected to provide a new theoretical basis and therapeutic target references for the early diagnosis, individualised treatment, and management of MI, thereby further improving patients' quality of life.

In summary, based on the transcriptome data of MI from the GEO database, this study integrates bioinformatics methods to screen hub genes related to MIT in MI, explore the association between MIT and MI, and analyse their potential as hub genes as well as the underlying molecular mechanisms. It is expected to provide a theoretical basis and technical support for the precise treatment of MI.

## 2 Results

### 2.1 MIT‐Related Candidate Genes Were Screened Out in MI

**FIGURE 1:**
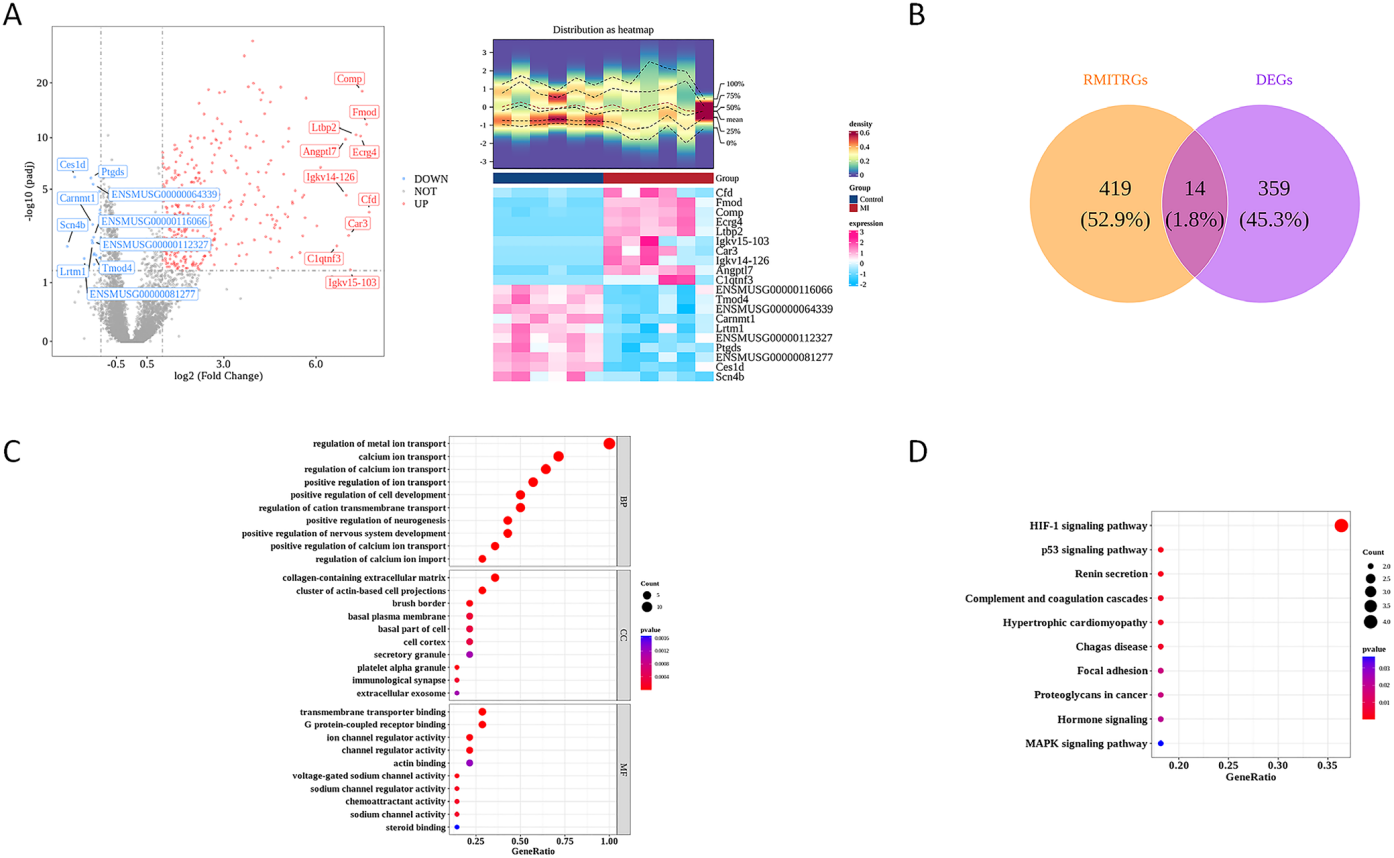
Screening of differentially expressed genes. (A) Volcano plot showing differentially expressed genes (GSE147365, adj. *p* < 0.05, |log_2_FC| > 1); (B) Heatmap of DEGs expression, along with heatmaps of TOP10 up–regulated and TOP10 down–regulated genes; (C) Venn diagram for identifying hub genes; (D) GO enrichment analysis.

The results of differential analysis showed that there were 373 DEGs in MI, among which 348 genes were up‐regulated, and 25 genes were down‐regulated (Figure 1A). In addition, 14 DEGs related to MIT were identified as candidate genes (Figure 1B). Enrichment analysis of these candidate genes revealed a total of 1203 significantly enriched related functions (*p* < 0.05), including 1059 biological process (BP) functions, 64 cellular component (CC) functions, and 80 molecular function (MF) functions. These functions are mainly involved in pathways such as regulation of metal ion transport, calcium ion transport, and regulation of calcium ion transport (Figure 1C, Supplementary Table S2). The KEGG enrichment results showed that the 14 candidate genes were enriched in 14 metabolic pathways (*p* < 0.05), mainly involving the HIF‐1 signalling pathway, p53 signalling pathway, Renin secretion signalling pathway, etc. (Figure 1D, Supplementary Table S3).

### 2.2 Determination of Hub Genes

**FIGURE 2:**
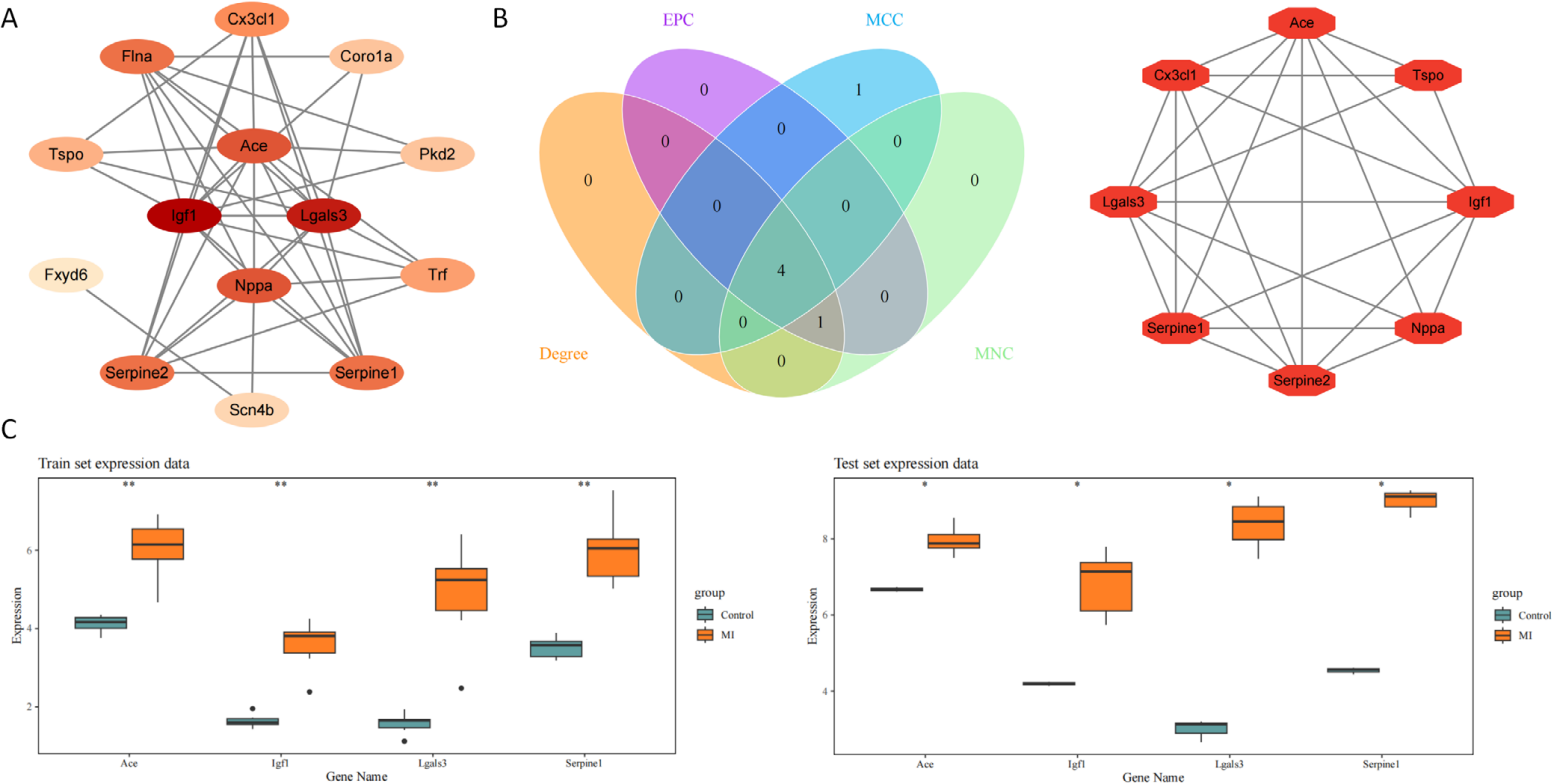
Screening of hub genes. (A) STRING database (https://cn.string‐db.org/) to analyse the interactions between 14 candidate gene‐related proteins and construct a PPI network (interaction score > 0.15); (B) Genes intersected by four algorithms of CytoHubba (left) and clusters of key functional modules identified by MCODE (right); (C) Expression of candidate hub genes in datasets of MI and control groups. Training set (left); Validation set (right). *p < 0.05, **p < 0.01, ***p < 0.001.

The Protein–protein interaction (PPI) network showed 82 interactions among 14 candidate gene‐related proteins, with insulin‐like growth factor 1 (Igf1), Galectin 3 (Lgals3), and angiotensin I converting enzyme (Ace) having high interaction degrees (Figure 2A). CytoHubba's four algorithms (MCC, EPC, MNC, Degree) identified 4 intersecting genes: Serpin family E member 1 (Serpine1), Lgals3, Ace, and Igf1. MCODE clustering found an 8‐gene up‐regulated module (MCODE score ≥ 5), which included these 4 genes, designated as characteristic candidates (Figure 2B). In GSE147365 and GSE236374, Ace, Igf1, Lgals3, and Serpine1 showed consistent up‐regulation in MI groups with significant differences vs. controls (*p* < 0.05) (Figure 2C), thus being identified as hub genes.

### 2.3 Hub Genes Are Involved in Multiple Related Signalling Pathways

**FIGURE 3:**
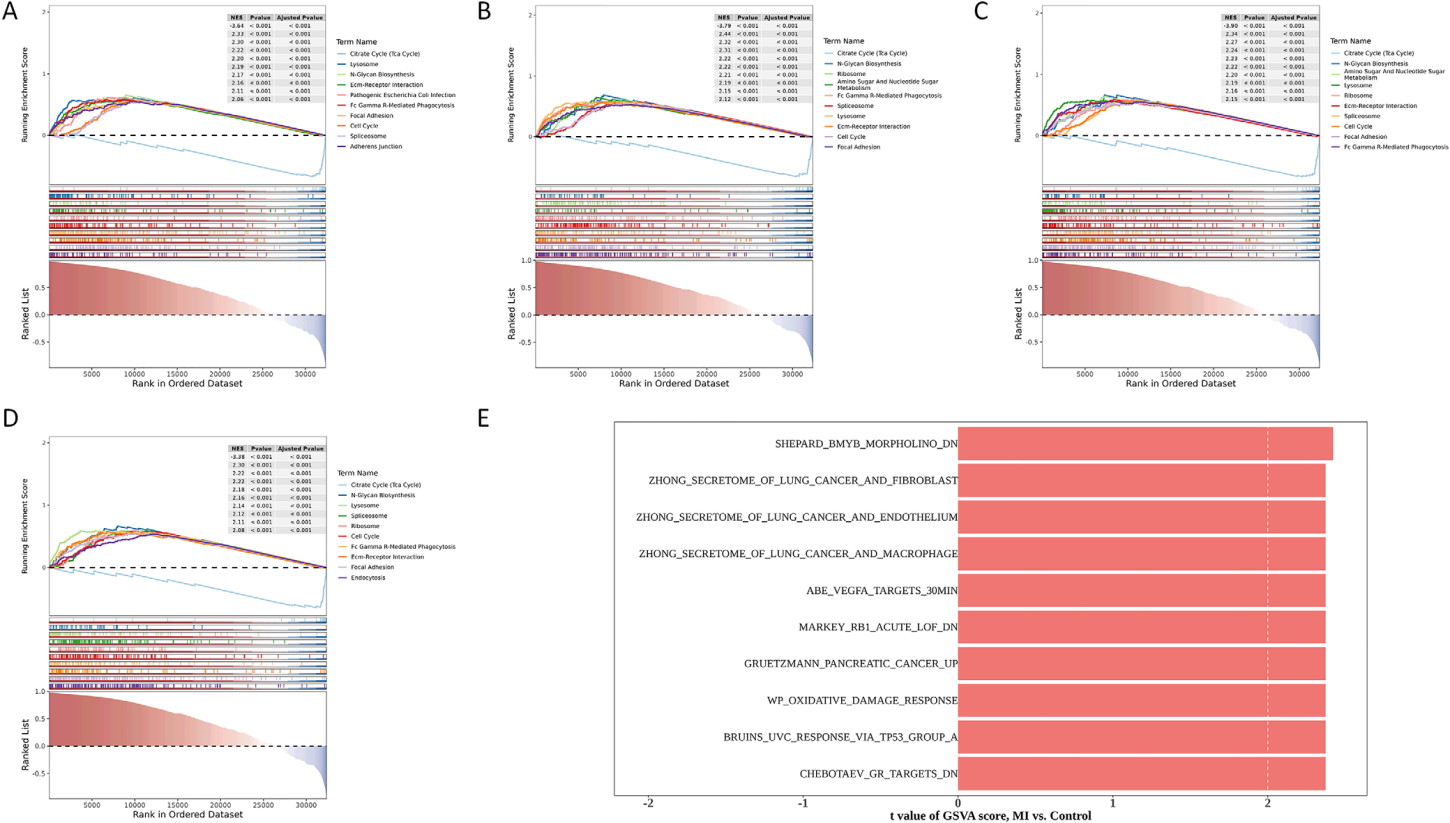
Hub genes are involved in multiple corresponding related signalling pathways. (A) GSEA analysis of Ace; (B) GSEA analysis of Igf1; (C) GSEA analysis of Lgals3; (D) GSEA analysis of Serpine1; (E) GSVA analysis of MI samples and control samples.

Gene Set Enrichment Analysis (GSEA) results for the four hub genes showed: Ace enriched in 124 pathways (e.g., Citrate Cycle, Lysosome, N‐Glycan Biosynthesis; Figure 3A, Supplementary Table S4); Igf1 in 129 pathways (e.g., Valine/Leucine/Isoleucine Degradation, Citrate Cycle; Figure 3B, Supplementary Table S5); Lgals3 (124 pathways) and Serpine1 (118 pathways) both significantly enriched in Citrate Cycle, N‐Glycan Biosynthesis, and Lysosome (Figure 3C,D, Supplementary Tables [Link], [Link]). These suggest they may contribute to MI via oxidative metabolism and cellular structure processes. Gene Set Variation Analysis (GSVA) identified 89 significantly different pathways between MI and control samples (Supplementary Table S8), mainly including “ZHONG SECRETOME OF LUNG CANCER AND MACROPHAGE” and “ZHONG SECRETOME OF LUNG CANCER AND ENDOTHELIUM” (Figure 3E), revealing regulatory roles of macrophages, endothelial cells, and tumour cells in MI‐related immune suppression, angiogenesis, invasion, and metastasis.

### 2.4 Correlation Between Hub Genes and Immune Cells

**FIGURE 4:**
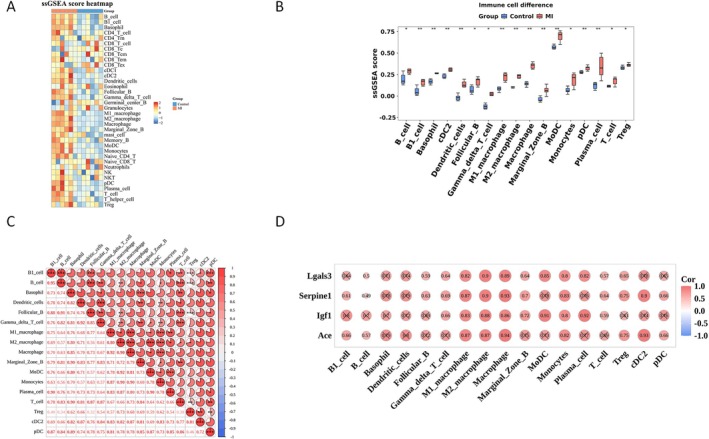
Hub genes exhibit significant correlations with immune cells. (A) Immune infiltration abundance: MI group vs. control group; (B) Differences in immune cell infiltration between the MI group and the control group; (C) Correlation analysis between immune cell subtypes; (D) Spearman correlation between hub genes and immune‐infiltrating cells. *p < 0.05, **p < 0.01, ***p < 0.001.

As shown in Figure 4A, the MI group had higher enrichment of “innate immune” cells (Neutrophils, Monocytes, M1 macrophages, Eosinophils) but lower “adaptive immune” cell scores (B cells, Follicular B cells, T cells, Gamma delta T cells) compared to controls. Seventeen cell types showed significant infiltration differences (*p* < 0.05), including B1 cells, Basophils, Dendritic cells, and M1 macrophages (Figure 4B). Correlation analysis revealed strong links (0.7–0.9) among B1 cells, macrophages/monocytes, and dendritic cells: For example, B1 cells vs. Follicular B cells (cor = 0.88, *p* < 0.001); M1 macrophages vs. M2 macrophages/Macrophages (cor > 0.8, *p* < 0.05) (Figure 4C). Hub genes Serpine1, Lgals3, Ace, and Igf1 had significant positive correlations with M1/M2 macrophages, macrophages, and monocytes (*p* < 0.05, cor ≥ 0.8). They also correlated strongly with Gamma delta T cells, T cells, and Treg cells (cor > 0.6, *p* < 0.05) (Figure 4D), suggesting these hub genes may regulate MI's immune microenvironment.

### 2.5 Prediction of Potential Drugs Targeting Hub Genes

**FIGURE 5:**
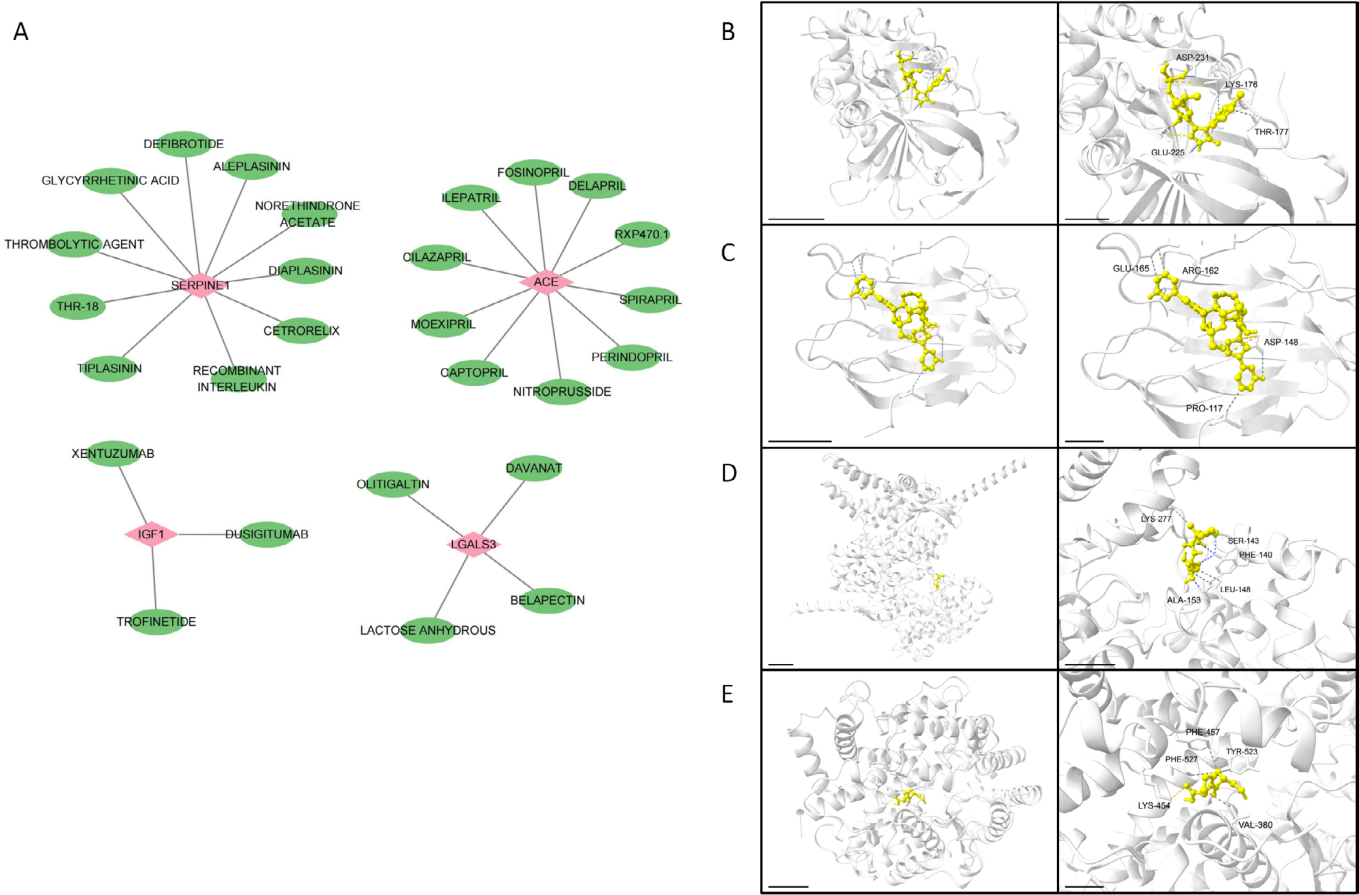
Drug prediction and molecular docking. (A) Drug prediction network diagram, illustrating the associations between hub genes and their predicted candidate drugs; (B) Molecular docking results of SERPINE1 with DEFIBROTIDE. (C) Molecular docking results of LGALS31 with OLITIGALTIN. (D) Molecular docking results of IGF1 with TROFINETIDE. (E) Molecular docking results of ACE with CAPTOPRIL. The protein backbone is shown in cartoon representation, the compounds in ball‐and‐stick representation, and the amino acid residues interacting with the compounds in stick representation. The dashed lines in the magnified view represent the predicted interactions between the compound and protein residues in CB‐Dock2: Blue for hydrogen bonds, light blue for weak hydrogen bonds, orange for ionic interactions, and grey for hydrophobic interactions.

Through gene homology analysis, the aforementioned hub genes were mapped to their corresponding human gene symbols. The results showed that Ace, Igf1, Serpine1, and Lgals3 were mapped to ACE, IGF1, SERPINE1, and LGALS3, respectively. As shown in Figure 5A, the top 10 drugs associated with each key human gene were predicted using the DGIdb database. Specifically, the ACE gene was associated with drugs such as DELAPRIL and ILEPATRIL; IGF1 was linked to DUSIGITUMAB, TROFINETIDE, and XENTUZUMAB; LGALS3 was correlated with DAVANAT and BELAPEATIN; while SERPINE1 was predicted to interact with CETRORELIX and TIPLASININ, among others. Subsequently, we further performed molecular docking simulations based on the structure of human proteins (Table 1). Specifically, the binding energy between SERPINE1 and DEFIBROTIDE was −6.6 kcal/mol, linked by hydrogen bonds with GLU‐225 and ASP‐231, as well as hydrophobic interactions with LYS‐176 and THR‐177 (Figure 5B). The binding energy between LGALS31 and OLITIGALTIN was −7.5 kcal/mol, connected via hydrophobic interactions with GLU‐165, ARG‐162, ASP‐148, and PRO‐117 (Figure 5C). The binding energy between IGF1 and TROFINETIDE was −8.0 kcal/mol, associated with hydrogen bonds involving SER‐143, PHE‐140, and ALA‐153, together with hydrophobic interactions with LYS‐277 and LEU‐148 (Figure 5D). The molecular docking of ACE with CAPTOPRIL (Figure 5E) showed a binding energy of −5.5 kcal/mol, with TYR‐523, VAL‐380, LYS‐454, PHE‐527 and PHE‐457 serving as the key binding sites. Notably, all binding energies were less than −5.0 kcal/mol, indicating good binding performance between the genes and corresponding drugs. These findings provide a reference for potential therapeutic drugs for myocardial infarction (MI).

**TABLE 1:** Molecular docking results.

ACE	Cavities_volume	Score(kcal/mol)
1	13,554	−5.8
2	239	−4.3
3	167	−5
4	130	−3.4
5	129	−4.2
SERPINE1	Cavities_volume	Score
1	295	−6.5
2	191	−6.5
3	189	−6.3
4	182	4.2
5	169	−6.4
IGF1	Cavities_volume	Score
1	8248	−7.7
2	6624	−7.7
3	2755	−6.5
4	1147	−6.1
5	872	−6.8
LGALS3	Cavities_volume	Score
1	122	−7.3
2	56	−6.9
3	55	−7.1
4	47	−6.7
5	44	−6.9

### 2.6 Regulatory Networks of Hub Genes and Associated Diseases

**FIGURE 6:**
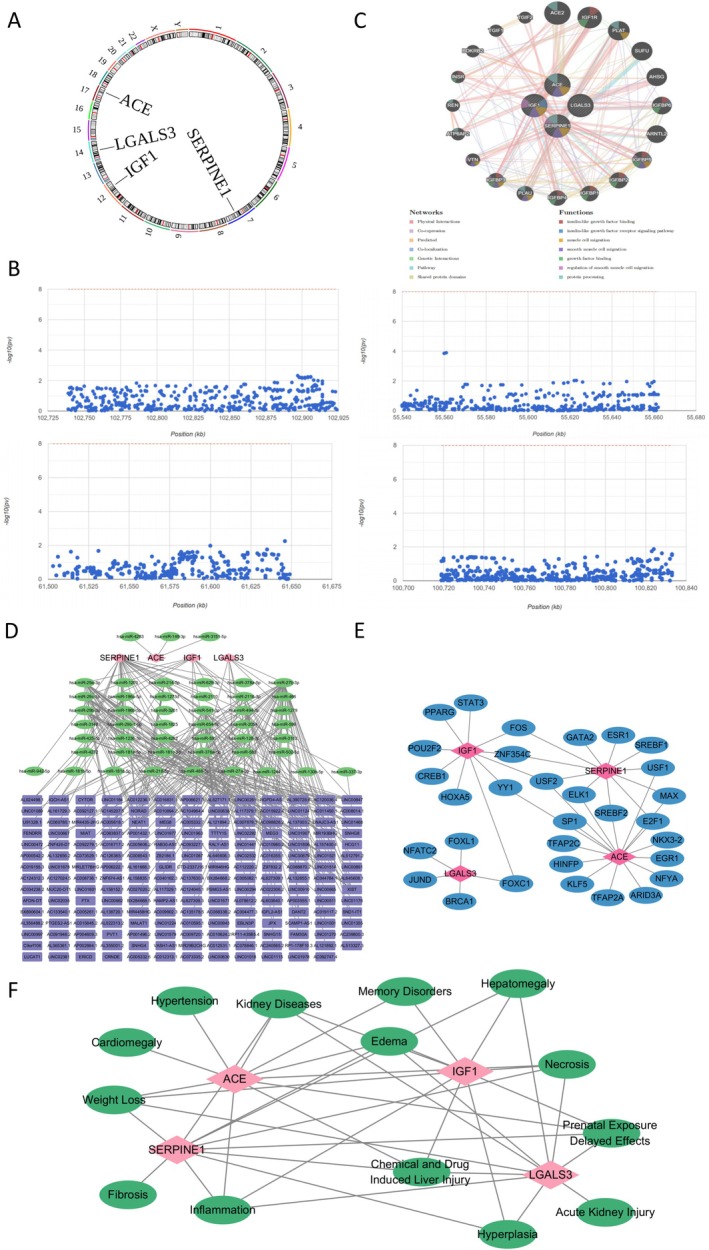
Regulatory networks of hub genes and their associated diseases. (A) Chromosomal localisation map of hub genes; (B) In the Gene Atlas database (http://geneatlas.roslin.ed.ac.uk/), GWAS data of MI were analysed to identify pathogenic regions of SNP loci associated with biomarkers; (C) GeneMANIA reveals the SNP interaction network of hub genes; (D) lncRNA‐miRNA‐hub gene regulatory network; (E) TFs‐mRNA regulatory network; (F) Disease prediction network diagram.

Chromosomal localisation showed IGF1 on chr12, LGALS3 on chr14, ACE on chr17, and SERPINE1 on chr7 (Figure 6A). MI GWAS data identified key SNPs for these genes, with no significant pathogenic variants in their regions (*p* < 5 × 10^−8^) (Figure 6B). In GeneMANIA, ACE, IGF1, SERPINE1, and LGALS3 were central, involved in functions like insulin‐like growth factor binding, muscle cell migration, and growth factor binding (Figure 6C). ENCori predicted 48 miRNAs targeting these genes: Three for ACE, 10 for IGF1, 7 for LGALS3, 28 for SERPINE1, interacting with 935 lncRNAs (e.g., lncRNA AC005332.7 regulates IGF1, LGALS3, SERPINE1 via specific miRNAs) (Figure 6D). In TF‐mRNA networks: ACE interacted with 16 TFs (e.g., SP1); IGF1 with 10 (e.g., USF2); LGALS3 with 5 (e.g., FOXC1); SERPINE1 with 11 (e.g., USF2). USF2 co‐targeted three genes, FOS/ZNF354C co‐targeted two, and FOXC1 co‐targeted two (Figure 6E). CTD database linked all four genes to seven diseases (Kidney Diseases, Necrosis, Inflammation, etc.) (Figure 6F), suggesting their involvement in these pathologies during MI.

### 2.7 Verification of the Hub Genes

**FIGURE 7:**
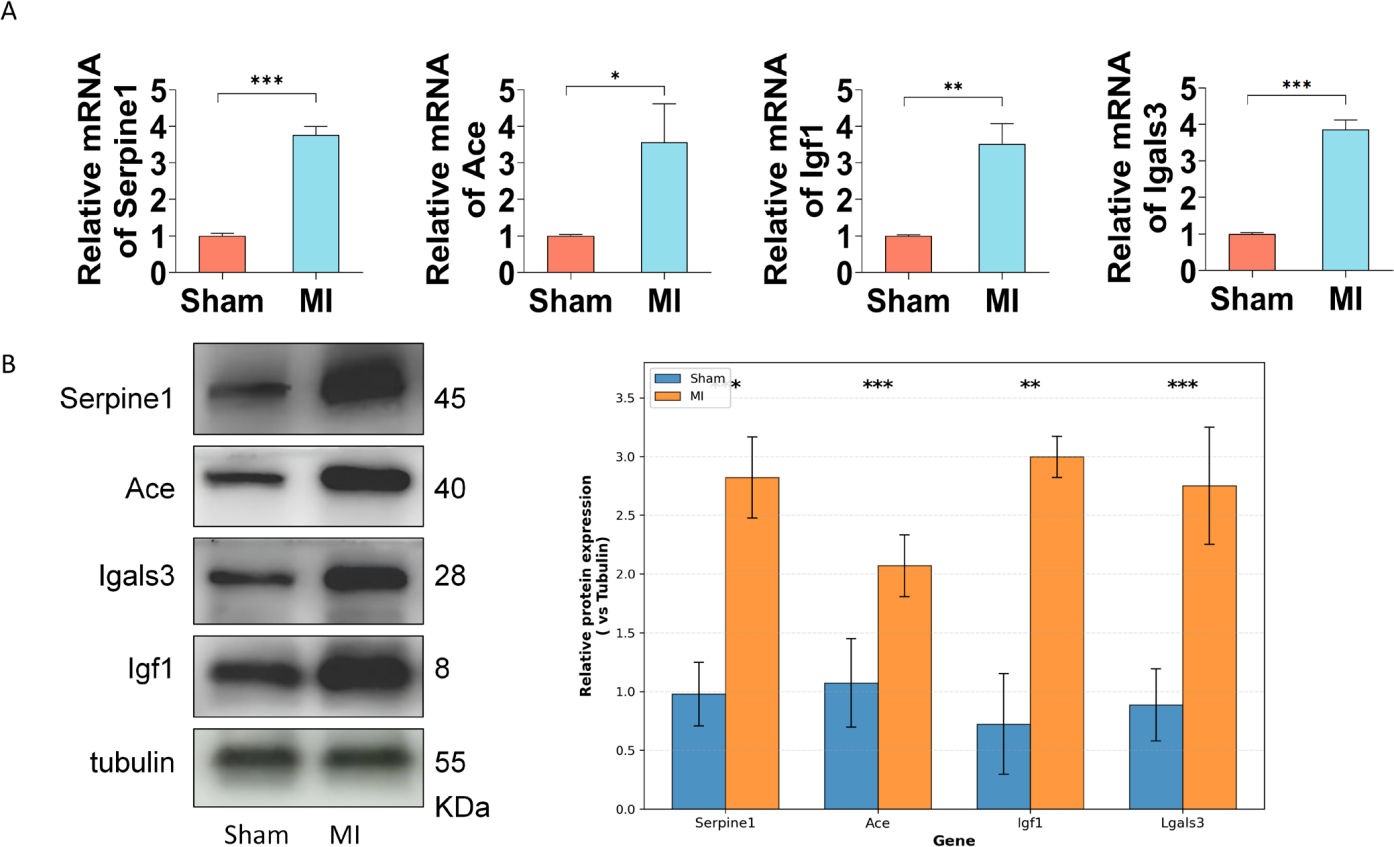
Validation of hub gene expression in MI mouse models. (A) qRT‐PCR analysis of Ace, Igf1, Lgals3, and Serpine1 mRNA expression levels in myocardial tissue from sham‐operated and MI mice (*n* = 5 per group). (B) Western Blotting analysis of ACE, IGF1, LGALS3, and SERPINE1 protein expression levels in myocardial tissue lysates (*n* = 3 per group). Tubulin was used as the loading control. Representative blots (left panel) and quantitative analysis (right panel) are shown. Data are presented as mean ± SEM. *p < 0.05, **p < 0.01, ***p < 0.001 compared to sham group.

The expression levels of the four hub genes (Ace, Igf1, Lgals3, and Serpine1) were validated in MI mouse models. qRT‐PCR analysis showed significantly elevated mRNA transcript levels for all four genes following MI modelling (Figure 7A). To further confirm these findings at the protein level, Western Blotting was performed on myocardial tissue lysates. Consistent with the transcriptional changes, the protein expression levels of ACE, IGF1, LGALS3, and SERPINE1 were significantly upregulated in the MI group compared to sham controls (Figure 7B), confirming that the hub genes are not only transcriptionally but also translationally activated during MI pathogenesis.

## 3 Discussion

Myocardial infarction (MI) is an acute and severe clinical manifestation of coronary artery disease, triggered by the acute occlusion of coronary arteries, and is characterised by high morbidity and mortality [13]. The homeostasis of MIT plays a crucial role in maintaining cellular functions and influencing the progression of various diseases, such as cardiovascular diseases (e.g., MI). Under conditions of myocardial ischemia and hypoxia, the disruption of MIT stability leads to a series of imbalances in signal transduction and subsequent cellular dysfunction [9]. However, the specific molecular mechanisms by which MIT contributes to MI pathogenesis remain unclear, particularly regarding the identity of involved hub genes and the specific biological pathways closely associated with MIT. To address this knowledge gap, this study used transcriptome data from the GEO database (GSE147365 as the training set and GSE236374 as the validation set) and employed a series of bioinformatics analyses to explore MIT‐related hub genes involved in MI. These mouse MIT‐related hub genes were then mapped to their corresponding human homologues to investigate the role of these human genes in MI pathogenesis, predict targeted therapeutic drugs, and identify diseases closely associated with these homologues—ultimately aiming to provide new references for MI treatment.

Serpine1, Lgals3, Ace, and Igf1 (all mouse homologues of human MIT‐related genes) are highly expressed in the MI group. Angiotensin‐Converting Enzyme (ACE, human homologue of mouse Ace) exists as a membrane‐bound form in endothelial cells, epithelial cells, and neuroepithelial cells (e.g., in the brain), and as a soluble form in blood and many body fluids [14]. Functionally, ACE is a key enzyme in the Renin‐Angiotensin System (RAS), catalysing the conversion of angiotensin I (Ang I) to angiotensin II (Ang II). The RAS plays a crucial role in blood pressure regulation and is recognised as an important pathogenic modulator in the pathogenesis of MI [15]. Human ACE can exacerbate myocardial ischemia, inflammation, and fibrosis through the RAS, making it a key molecule in MI progression [16]. Furthermore, existing studies have shown that human ACE is expressed in cardiac endothelial cells and renal tubular epithelial cells. Consistently, in animal models, mice with Ace deficiency (mouse Ace, homologue of human ACE) exhibit gradual impairment of cardiac function, while Ace transgenic mice develop ventricular tachycardia and cardiac conduction block [17]. These findings—from both human cell studies and mouse models—highlight the role of ACE in cardiac remodelling and heart failure. Human IGF1 serves as the primary mediator of growth hormone (GH) effects and acts as a key regulator of cell growth, differentiation, and apoptosis [18]. As a polypeptide hormone secreted by the liver, IGF1 exerts its functions by activating the IGF1 receptor (IGF1R) and is involved in the regulation of metabolism, cell survival, and other cellular processes relevant to cardiovascular homeostasis [19]. Studies have revealed that beyond its known growth‐promoting and metabolic effects, IGF1 plays specific roles in the complex cascade of cardiovascular physiological and pathological functions. Notably, it can promote cardiac growth, improve cardiac contractility, enhance cardiac output and ejection fraction, thereby exerting beneficial effects on MI [20]. In the cardiovascular system, IGF1 can activate the PI3K/Akt pathway to inhibit myocardial cell apoptosis [21]. Consistent with these findings, the present study identified the mouse Igf1 gene (human IGF1 homologue) as an MIT‐related hub gene in MI; this mouse Igf1 gene is also enriched in metabolic pathways such as the citric acid cycle and amino acid degradation. By inhibiting tissue‐type plasminogen activator (tPA) and urokinase‐type plasminogen activator (uPA), SERPINE1 impairs plasmin generation, reduces fibrin degradation, and thereby promotes thrombus formation [22]. The heart can self‐repair after MI through post‐MI fibrotic responses, where the extent of fibrosis is determined by the balance between extracellular matrix deposition secreted by activated fibroblasts and the breakdown of newly formed scar tissue by proteases secreted by inflammatory cells [23]. As the largest and most functionally diverse member of the evolutionarily conserved serpin family (a family of protease inhibitors), human SERPINE1 can regulate matrix functions and post‐MI cardiac remodelling [24]. Since MI is often triggered by thrombotic occlusion in coronary arteries, the high expression of SERPINE1 may increase thrombotic risk [22], which is consistent with the findings of our study. Human LGALS3 is a β‐galactoside‐binding protein belonging to the lectin family, which regulates various physiological and pathophysiological processes in the human body [25]. It is primarily involved in inflammation, fibrosis, and tissue repair, and is closely associated with the occurrence, development, and prognosis of MI [26]. LGALS3 has been linked to the pathogenesis of several diseases, including organ fibrosis, chronic inflammation, cancer, atherosclerosis, and other cardiovascular diseases [27]. Animal models have demonstrated that inhibition of LGALS3 can reduce myocardial fibrosis and improve cardiac function, suggesting that it may serve as a novel target for anti‐myocardial remodelling strategies [28]. Similarly, the present study indicates that Lgals3 can act as a hub gene.

The mouse MIT‐related hub genes are primarily enriched in pathways such as the Citrate Cycle (TCA Cycle), Lysosome‐related pathway, and N‐Glycan Biosynthesis. Among these, the Citrate Cycle (TCA Cycle) serves as a metabolic hub for carbohydrates, lipids, and amino acids—particularly cardiomyocytes—linking catabolism and anabolism. Studies have revealed that succinate, an intermediate of the citrate cycle, accumulates during myocardial ischemia and generates reactive oxygen species (ROS) through reverse electron transport, thereby exacerbating myocardial damage [29]. Furthermore, cardiomyocytes possess the highest mitochondrial content in the heart and, due to their high energy demand for sustaining cardiac contraction, are more vulnerable to energy depletion and oxidative stress compared to non‐cardiomyocytes. Mitochondrial function is a core feature of cardiac health and a critical component in the pathogenesis of many cardiovascular diseases, including MI [30]. In myocardial tissue, impairment of citrate cycle (TCA cycle) activity—caused by blocking 2‐oxoglutarate dehydrogenase—leads to a rapid decline in cardiac contractile function, but this phenomenon can be reversed by adding anaplerotic substrates that promote carboxylase flux (e.g., malic enzyme, pyruvate carboxylase, and malonyl‐CoA carboxylase) [31]. In the pathological process of MI, lysosomes play a dual role: They are involved in cardiomyocyte death (e.g., apoptosis/necrosis caused by lysosomal membrane permeabilisation) while simultaneously maintaining cellular homeostasis through the activation of protective autophagy [32]. Under physiological conditions, lysosomes degrade cytoplasmic components via autophagy, including individual proteins, protein aggregates, and defective or excess organelles. Autophagy eliminates abnormal or surplus organelles [33]. Furthermore, N‐Glycan Biosynthesis—a process by which carbohydrates (glycans) are covalently attached to proteins and that modulates protein function—is one of the most common post‐translational modifications of proteins [34]. Dysregulation of N‐glycan biosynthesis contributes to multiple pathological processes in MI, such as inflammatory responses, thrombosis, cardiomyocyte apoptosis, and impaired myocardial repair. For example, abnormal N‐glycosylation increases fibrin clot density and delays fibrinolysis, thereby promoting coronary artery thrombosis—a direct trigger of acute MI [35]. In the heart, dysregulation of protein glycosylation contributes to the pathophysiology of heart failure and cardiac hypertrophy—two common sequelae of MI. Meanwhile, dysregulated protein glycosylation in the vascular system promotes changes in cell–cell interactions, inflammation, and vascular remodelling, including atherosclerosis, a key risk factor for MI [36].

Immune infiltration analysis revealed significant differences in immune cell populations between the mouse MI group and the mouse control group, with significant differential infiltration of neutrophils, monocytes, M1 macrophages, and dendritic cells. Moreover, these differentially infiltrated immune cells were significantly correlated with the four mouse MIT‐related hub genes. Based on this correlation, it can be predicted that the hub genes may further influence the behaviour and functions of immune cells, thereby regulating the immune microenvironment in mouse MI. For instance, overactivated neutrophils can promote a state of chronic inflammation and drive the initiation of innate and adaptive immune responses, whereas depletion of neutrophils during post‐MI cardiac repair exacerbates the development of heart failure [37]. Studies have found that the number of cardiac monocytes and macrophages increases rapidly during the acute phase of myocardial infarction [38]. Furthermore, in both the acute and chronic phases after myocardial infarction in mice, the production of monocytes in the bone marrow and extramedullary sites is regulated by sympathetic nerve signals and IL‐1 [39].

The human homologues of the four mouse MIT‐related hub genes exhibit strong binding affinity for their corresponding drugs, as indicated by binding energies < −5 kcal/mol in our molecular docking simulations. For example, delapril—a drug targeting human ACE (mouse Ace homologue)—has shown favourable efficacy in improving the signs and symptoms of congestive heart failure with good tolerability (a common complication of MI) and is well‐tolerated [40]. Meanwhile, belapectin—a galectin‐3 (human LGALS3, mouse Lgals3 homologue) inhibitor—primarily improves metabolic dysfunction in patients with cirrhosis and portal hypertension; preclinical studies further suggest it may reduce post‐MI myocardial fibrosis (consistent with LGALS3's role as an MI‐related hub gene) [41]. In addition, disease prediction identified seven conditions associated with the human homologues of the hub genes: Kidney diseases, necrosis, inflammation, oedema, weight Loss, chemical and drug‐induced liver injury, and prenatal exposure delayed effects. During MI, multiple cell death programs are activated, including mitochondria‐dependent necrosis and apoptosis; however, deletion of BAK, a pro‐death protein, reduces MI size to a certain extent—consistent with our finding that hub genes (e.g., Igf1) regulate myocardial cell apoptosis [42]. Consistent with our CTD‐based disease prediction that kidney diseases are associated with the hub genes, studies have found that acute myocardial infarction is a leading cause of death in chronic kidney disease (CKD) patients undergoing haemodialysis. During CKD, immune dysfunction and metabolic changes—such as elevated levels of inflammatory cytokines, disrupted lipid and mineral ion homeostasis (a process closely linked to MIT, the core focus of our study), and accumulation of uremic toxins—exacerbate the instability of atherosclerotic plaques and promote vascular calcification, which are key pathophysiological mechanisms driving MI development. Furthermore, haemodialysis itself exerts adverse effects on lipoproteins, the immune system, and haemodynamics—effects that may further interact with the hub genes to exacerbate MI susceptibility in CKD patients [43].

In this study, by integrating Bulk‐RNA‐seq transcriptome data and multiple bioinformatics approaches, we systematically identified MIT‐related hub genes in MI: Ace, Igf1, Serpine1, and Lgals3. Immune infiltration correlation analysis revealed that these mouse hub genes are significantly correlated with macrophages, monocytes, and dendritic cells in MI, suggesting that they may possess specific functions in immune cell differentiation, further supporting their potential role in the regulation of immune microenvironment homeostasis in MI. Despite obtaining relatively comprehensive findings, this study still has several notable limitations that need to be addressed. First, the study is based on data from mouse models, and the diagnostic efficacy of the hub genes has not yet been validated in human clinical samples. The biological functions of the hub genes and their specific mechanisms in MI still require experimental validation. In this study, we validated the expression of the hub genes at both the transcriptional and protein levels using qRT‐PCR and Western Blotting in MI mouse models. To further explore their functional roles, subsequent studies will employ ELISA to quantify secretory protein concentrations in myocardial tissue homogenates and peripheral blood, and IHC/IF techniques to localise protein expression distribution within cardiac tissue. Integrating multi‐omics approaches, including proteomics and metabolomics, will help comprehensively dissect the molecular regulatory network of MI and facilitate the development of targeted therapeutic strategies. Additionally, developing targeted drugs or intervention strategies based on these hub genes will promote the precision treatment of MI.

## 4 Conclusion

Using transcriptome data, this study systematically analysed the transcriptomic characteristics of MI patients and screened for MIT‐related hub genes, ultimately identifying four critical genes: ACE, IGF1, SERPINE1, and LGALS3. The findings of this study provide a new research direction for the targeted therapy of MI. Future studies will require further experimental and clinical validation to advance its translational application.

## 5 Materials and Methods

### 5.1 Data Sources

The training dataset GSE147365, obtained from NCBI's GEO database with sequencing platform GPL24247, includes 6 cardiac tissue samples from mice with MI and 6 from sham‐operated controls. For validation, dataset GSE236374 from GEO (same platform) was used, containing 6 MI samples (3 at 7 days, 3 at 28 days post‐MI) and 3 sham controls. Additionally, 433 metal ion transport‐related genes (MITRGs) were screened from the GOBP_REGULATION_OF_METAL_ION_TRANSPORT gene set via MSigDB.

### 5.2 Identification of Differentially Expressed Genes

Differential expression analysis of MI vs. control groups was performed using the R package “DESeq2” (v3.20.0) on training set GSE147365 to identify differentially expressed genes (DEGs), with screening criteria: Adjust. *p* < 0.05 and |log2 FC| > 1. A volcano plot (via “ggplot2” v3.5.1) showed overall DEG distribution, labelling the top 10 up/downregulated genes by |log2 FC|. A heatmap (“Heatmap” v1.0.12) illustrated these genes' expression patterns in both groups.

### 5.3 Identification and Functional Analysis of Candidate Genes

Using the R package “ggvenn” (v0.1.10), intersection analysis of DEGs and MITRGs identified overlapping candidate genes. R packages “clusterProfiler” (v4.10.1) and “org.Mm.eg.db” (v3.21.0) were used for GO (CC, BP, MF) and KEGG enrichment analyses (*p* < 0.05), revealing immune pathways and signalling mechanisms. Top 10 pathways for each GO category and KEGG (by ascending *P*‐values) were visualised.

### 5.4 Identification of Hub Genes

To investigate potential protein–protein interaction relationships among the candidate genes, a PPI network was constructed using the Search Tool for the Retrieval of Interacting Genes (STRING) database (https://cn.string‐db.org/), with an interaction confidence threshold set to interaction score > 0.15. The results were visualised using Cytoscape software (version 3.10.0). Based on the PPI network, the Cytoscape plugin MCODE (Molecular Complex Detection) was used to identify functionally closely related protein interaction modules. The parameters were set as follows: MCODE score ≥ 5, degree cutoff = 2, node score cutoff = 0.2, k‐core = 2, and max depth = 100. Subsequently, the Cytoscape plugin CytoHubba (version 3.10.0) was used to score the importance of each node in the PPI network. Four algorithms—Maximal Clique Centrality (MCC), Edge Percolated Component (EPC), Maximum Neighbourhood Component (MNC), and Degree Centrality (Degree)—were employed to rank and score the candidate genes. The intersection of the top 5 genes from each algorithm was identified using the R package “ggvenn” (version 0.1.10) to screen for genes with consistently high scores across multiple algorithms. Finally, the highest‐scoring module genes from MCODE and the intersecting genes from CytoHubba were integrated: Intersecting genes included in the MCODE module were retained, and their positions within the module were annotated to reveal their regulatory roles in the local network, designated as characteristic candidate genes. To evaluate the expression differences of the screened characteristic candidate genes between MI samples and healthy control samples, boxplots were generated using the R packages “ggplot2” (version 3.5.1) and “ggpubr” (version 0.6.0) for both the training set GSE147365 and the validation set GSE236374 Statistical significance of expression differences was assessed using the Wilcoxon rank‐sum test, with a significance threshold of *p* < 0.05. Genes showing significant differences with consistent expression trends across all datasets were ultimately defined as hub genes.

### 5.5 GSEA And GSVA

To explore KEGG pathway differences between MI and control samples, GSVA was performed on training set GSE147365 using “GSVA” (v1.50.5) to calculate signalling pathway enrichment scores, with reference gene set “c2.all.v7.5.1.symbols.gmt” from MSigDB. Pathway differences were compared via “Limma” (v3.58.1) with criteria: |t| > 2, *p* < 0.05, |log FC| > 0.5. To investigate hub genes' biological functions in MI pathogenesis, GSEA was conducted. “Psych” (v2.4.3) calculated Pearson correlations between each hub gene and all training set genes, generating ranked lists by descending coefficients. Relevant mouse gene sets from the GSEA official website (https://www.gsea‐msigdb.org/gsea/msigdb/mouse/collections.jsp) were downloaded via custom code, and GSEA on these lists used “clusterProfiler” (v4.10.1) with criteria: Adjust. *p* < 0.05, |NES| > 1. Results were visualised with “enrichplot” (v1.22.0).

### 5.6 Immune Infiltration Analysis

To explore immune microenvironment differences between MI and control samples, ssGSEA (via GSVA v1.50.5) evaluated infiltration levels of 36 mouse immune cell types using GSE147365. Wilcoxon tests (*p* < 0.05) identified differentially infiltrated cells, whose infiltration levels were correlated (|cor| > 0.3, *p* < 0.05) and visualised in a heatmap to show synergistic/antagonistic relationships. Hub genes' immune regulatory roles were analysed via Spearman correlation (“psych” v2.4.3) with differentially infiltrated cells (|cor| > 0.3, *p* < 0.05), revealing their potential role in MI immune imbalance via specific cell subsets.

### 5.7 Drug Prediction and Molecular Docking

To explore the potential hub genes' pharmacological intervention potential, in silico prediction of small‐molecule drugs and molecular docking with human proteins were performed. Mouse hub genes were mapped to human homologues via the MGI database homology analysis. Drug‐gene interactions were mined using DGIdb, with the top 10 (or all) candidate drugs visualised in Cytoscape (v3.10.0) by interaction score. For structural binding evaluation, FDA‐approved or Phase II drugs (by score) were selected. Small‐molecule SDF structures (PubChem) and human protein PDB structures (PDB) were retrieved for molecular docking via CB DOCK2 (binding energy < −5 kcal/mol indicates high affinity). These human‐based analyses preliminarily explored the theoretical pharmacological regulation potential of MI hub genes; clinical applicability requires further validation, with higher total scores indicating greater receptor‐ligand affinity in stable conformations.

### 5.8 Construction of Regulatory Networks

To explore regulatory mechanisms of human hub genes in MI progression, multi‐component networks involving non‐coding RNAs and transcription factors were analysed. miRNAs targeting hub genes were predicted via miRanda and TargetScan; their intersection (hub miRNAs) was identified using “ggvenn” (v0.1.10). ENCORI predicted lncRNAs regulating these miRNAs, and a lncRNA‐miRNA‐hub gene network was visualised in Cytoscape (v3.10.0). NetworkAnalyst predicted transcription factors (TFs) for hub genes, with a TF‐hub gene regulatory network visualised in Cytoscape (v3.10.0).

### 5.9 Chromosomal Localisation, Genome‐Wide Association Study, and GeneMANIA Network Construction

Gene chromosomal localisation analysis (via “RCircos” v1.2.2) determined positions of human hub genes. MI‐related GWAS data from The Gene Atlas were analysed to identify SNP pathogenic regions in hub genes, with results visualised as Manhattan plots including key SNPs. A gene interaction network was constructed via GeneMANIA, which integrates co‐expression, protein interactions, and pathway data to predict functional synergies between hub genes and their neighbours, visually revealing their functional links and biological roles in MI progression.

### 5.10 Disease Prediction

To understand the relationship between human hub genes and other diseases, and to explore MI pathogenesis and potential therapeutic strategies, the Comparative Toxicogenomics Database (CTD, http://ctdbase.org/)—a robust public database for predicting gene/protein‐disease associations, integrating chemical‐disease, chemical‐gene, and gene‐disease interactions to predict novel associations and generate extended networks—was used. Hub genes were input into the CTD database to identify their most relevant diseases, with the top 10 results ranked by Inference score displayed. Network diagrams for each gene were generated using the R package “cytoscape” (version 3.10.0).

### 5.11 Animals and Treatment

Eight‐week‐old C57 mice, with a balanced sex ratio (50% male and 50% female), were obtained from SLACCAS (Hunan, China). The animals were kept at 25°C under conventional lighting conditions. They were randomly assigned to two groups: The experimental group received LAD ligated using a 7–0 nylon suture. Sham‐operated mice underwent the same procedure without ligation. All animal care and experimental protocols complied with animal welfare ethical standards and were approved by the Ethics Committee of the Experimental Animal Center at Central South University (Hunan, China; approval no. 202408136). The study was conducted in line with the ARRIVE guidelines. At corresponding time points after model establishment, cardiac function parameters were examined by echocardiography. The left ventricular ejection fraction (LVEF) of mice in the myocardial infarction (MI) group was less than 50% and was significantly reduced by ≥ 20% compared with the sham‐operation group, and the left ventricular fractional shortening (LVFS) was also significantly decreased. Meanwhile, combined with electrocardiographic findings, mice in the MI group exhibited typical ST‐T segment changes. The successful establishment of the MI model was determined based on the above two characteristic indicators [44].

### 5.12 Quantitative Reverse Transcription Polymerase Chain Reaction (qRT‐PCR)

Peripheral blood samples were collected from the mice, and total RNA (2 μg) was isolated using TRIZOL reagent (Invitrogen, CA, USA). First‐strand cDNA was synthesised according to the manufacturer's protocol (Takara Biotechnology, Shiga, Japan). qRT‐PCR was performed using the SYBR Green system (Takara Biotechnology) (primers were shown in Table S1). Primer sequences were produced by Sangon Biotechnology Co. Ltd. (Shanghai, China). mRNA expression levels were calculated using the 2^−△△Ct^ method.

### 5.13 Western Blotting

Myocardial tissue samples from the infarct zone were lysed in RIPA buffer (Beyotime, China) containing protease inhibitor cocktail (Roche, Switzerland). Protein concentrations were determined using the BCA protein assay kit (Thermo Fisher Scientific, USA). Equal amounts of protein (30 μg) were separated by 10% SDS‐PAGE and transferred onto PVDF membranes (Millipore, USA). Membranes were blocked with 5% non‐fat milk in TBST for 1 h at room temperature and then incubated overnight at 4°C with primary antibodies against ACE (1:1000, Abcam, ab254222), IGF1 (1:500, Santa Cruz, sc‐518,040), LGALS3 (1:1000, Cell Signalling Technology, #87985), SERPINE1 (1:500, Santa Cruz, sc‐52,979), and GAPDH (1:5000, Proteintech, 60,004–1‐Ig). After washing, membranes were incubated with HRP‐conjugated secondary antibodies for 1 h at room temperature. Protein bands were visualised using an ECL chemiluminescence detection kit (Bio‐Rad, USA) and quantified by ImageJ software (NIH, USA). Tubulin was used as the internal loading control.

### 5.14 Statistical Analysis

All analyses were performed using R software (version 4.5.1). Statistical significance was defined as *p* < 0.05. Differences between groups were compared using the Wilcoxon rank‐sum test.

## Author Contributions

**Xiaowei Zhou:** writing – review and editing, conceptualization, methodology. **Fanyan Luo:** writing – review and editing, validation. **Yao Deng:** writing – original draft, software, data curation. **Meihong Yu:** writing – review and editing, validation.

## Funding

This work was supported by the Hunan Provincial Natural Science Foundation of China (No. 2025JJ60803).

## Consent

All authors have given their consent for publication.

## Conflicts of Interest

The authors declare no conflicts of interest.

## Supporting information


**Table S1:** Primers for qRT‐PCR.

**Table S2:** GO analysis of these candidate genes.

**Table S3:** KEGG enrichment results.

**Table S4:** GSEA results for the four hub genes.

**Table S5:** Igf1 (129 pathways).

**Table S6:** Lgals3 (124 pathways).

**Table S7:** Serpine1 (118 pathways).

**Table S8:** GSVA pathways between MI and control samples.

## Data Availability

The data that support the findings of this study are available from the corresponding author upon reasonable request.
